# Cadmium Sources and Toxicity

**DOI:** 10.3390/toxics7020025

**Published:** 2019-05-06

**Authors:** Soisungwan Satarug

**Affiliations:** Kidney Disease Research Collaborative, Faculty of Medicine and Translational Research Institute, The University of Queensland, 37 Kent Street, Woolloongabba, Brisbane 4102, Australia; sj.satarug@yahoo.com.au

This special issue of Toxics, Cadmium (Cd) sources and toxicity, consists of one comprehensive review [1], three epidemiologic investigations [2,3,4] and five laboratory-based investigations [5,6,7,8,9].

A review article highlights environmental exposure to Cd and its association with chronic kidney disease (CKD) together with data from total diet studies (TDS) in which Cd was found to be present in virtually all foodstuffs [1]. Consequently, foods that are frequently consumed in large quantities such as rice, potatoes, wheat, leafy salad vegetables and other cereal crops are the most significant dietary Cd source [1]. Cd levels found in human livers and kidneys are provided together with current standards for tolerable intake, the urinary threshold of Cd and the utility of urinary Cd excretion as a measure of body burden of Cd.

In a cross sectional study of 395 Thai subjects [2], an inverse association was observed between urinary excretion of β_2_-microglobulin (β_2_MG) and estimated glomerular filtration rate (eGFR) simultaneously with an increase in the prevalence odds of low GFR (eGFR < 60 mL/min/1.73 m^2^) in subjects with an elevation of β_2_MG excretion, indicative of tubular dysfunction. Thus, a sign of Cd toxicity (tubular dysfunction) was linked to GFR reduction, and an increased risk of CKD, defined as eGFR < 60 mL/min/1.73 m^2^. These findings suggest that tubular pathology may have caused nephron atrophy and GFR loss [10].

In a 26-year follow-up study of 7,348 residents of the Jinzu River basin in Toyama, a highly polluted area of Japan [3], a 1.49-fold increase in deaths from cancer was observed in women who showed, 26 years earlier, signs of Cd-related kidney pathologies, such as proteinuria and glycosuria. The specific cancer types were uterus, kidney, kidney plus urinary tract. Paradoxically, in men, the risk of lung cancer and the risk of dying from malignant disease were reduced.

In a Chinse cohort study of 429 women, moderate and high environmental Cd exposure levels were associated with an early menarche [4]. Levels of environmental exposure as low, moderate or high were based on rice Cd concentration of 0.07, 0.51 and 3.7 mg/kg, respectively. The age of menopause in three areas did not differ. However, there were 1.3-and 3.7-fold increases in the likelihood of having menarche at age below 13 years in respectively moderate- and high-Cd exposure areas, compared with a low-exposure area. This Chinese finding is consistent with an early onset of puberty seen in Cd-treated female rats [11], thereby suggesting Cd has estrogenic activity.

Effects of inhaled Cd on developing kidneys were examined in Wistar rats [5]. In this study, Cd was administered to pregnant rats from gestation day 8 to day 20 via inhalation of CdCl_2_ aerosol (17.43 mg/m^3^) for 2 hours per day. This procedure delivered a dose of 1.48 mg Cd^2+^/kg/day. Pregnant rats inhaled normal saline aerosol served as controls. Kidneys from fetuses at gestation day 21 were examined for DNA binding activity of the transcription factor, hypoxia-inducible factor 1 (HIF-1). HIF-1 plays a critical role in the regulation of oxygen consumption, cell survival, growth and development. HIF-1 from kidneys of fetuses of Cd-intoxicated dams showed impairment in DNA-binding activity concomitant with reduced transcript levels for vascular endothelial growth factor (VEGF), one of the HIF-1 regulated genes. However, a compensatory mechanism was apparent as the VEGF protein abundance remained unchanged. These findings suggest potential effects of inhaled Cd on developing kidneys.

Effects of Cd on mature kidneys were examined in male Sprague-Dawley rats [6]. Kidney injury, reflected respectively by 2.2-, 21.7-, and 6.1-fold increases in urinary protein, KIM-1 and β_2_MG levels were induced after subcutaneous injections of CdCl₂ (0.6 mg/kg) 5 days a week for 12 weeks. Accompanied these urinary indictors of kidney effects were altered expression levels of microRNA (miRNA) in kidney cortex; levels of 44 miRNAs were increased, while levels of another 54 miRNAs were decreased. Thus kidney injury by Cd occurred concurrently with dysregulated miRNA expression in the rat renal cortex. These findings implicated miRNA as mediators of Cd-induced kidney injury.

Effects of Cd on periodontal bone were investigated in male Sprague-Dawley rats, given daily subcutaneous injections of Cd (0.6 mg/kg/day) 5 days a week for 12 weeks [7]. The distance between the cementoenamel junction and the alveolar bone crest was greater in Cd-intoxicated rats than controls. This was taken as evidence for Cd as a possible contributing factor to periodontal disease, thereby explaining an association between elevated body content of Cd and an increased risk of periodontal disease seen in the representative U.S. population.

Effects of Cd on mitochondria were examined in the INS-1 human pancreatic β-cell line [8]. Cd concentration ten-fold below the level causing cell death produced no effects on mitochondrial function, assessed with the energy charge and the synthesis of adenosine triphosphate (ATP). This Cd concentration, however, caused mitochondrial morphological change toward circularity, indicative of fission. The increased circularity suggested mitochondrial adaptive response to low-level Cd. If cellular Cd influx continues, impairment of this organelle may contribute to cellular dysfunction and decreased viability of β-cells, as seen in diabetes.

Therapeutic actions of the anti-diabetic drug, metformin were examined in male Wistar rats given Cd in drinking water (32.5 ppm) alone or Cd plus metformin (200 mg/kg/day) [9]. Cd treatment was found to cause hyperinsulinemia, insulin resistance, adipocyte dysfunction, loss of hepatic insulin sensitivity. Progressive accumulation of triglycerides was also seen in various tissues, while glycogen deposits were diminished in liver, heart, and renal cortex, but was increased in the muscle. Metformin showed a limited therapeutic efficiency on glucose tolerance and lipid accumulation that were induced by Cd.

In summary, this collection of research articles provides an update of knowledge on adverse effects of environmental Cd exposure, such as increased mortality from cancer, especially in women [3], an early menarche onset [4] and an increased risk of chronic kidney disease [2]. Potential effects of inhaled Cd on the development of kidneys in fetuses were evident in a study using pregnant Wistar rats [5]. Work with Sprague-Dawley rats suggested that dysregulation of a range of miRNAs mediated renal Cd toxicity [6] and that Cd contributed to periodontal disease [7]. An early effect of low-dose Cd on mitochondria in human pancreatic β-cells was observed [8]. However, therapeutic efficiency of metformin was not demonstrable when the drug was given to the Wistar rats with Cd-induced metabolic derangements [9].
